# Ursolic Acid Induces Apoptosis in Colorectal Cancer Cells Partially via Upregulation of MicroRNA-4500 and Inhibition of JAK2/STAT3 Phosphorylation

**DOI:** 10.3390/ijms20010114

**Published:** 2018-12-29

**Authors:** Karam Kim, Eun Ah Shin, Ji Hoon Jung, Ji Eon Park, Dong Soub Kim, Bum Sang Shim, Sung-Hoon Kim

**Affiliations:** College of Korean Medicine, Kyung Hee University, Seoul 02447, Korea; enpro21@naver.com (K.K.); eunah1008@khu.ac.kr (E.A.S.); johnsperfume@khu.ac.kr (J.H.J.); wdnk77@naver.com (J.E.P.); dongsoub@hanmail.net (D.S.K.); eshimbs@khu.ac.kr (B.S.S.)

**Keywords:** ursolic acid, miR-4500, colorectal cancer, apoptosis, STAT3

## Abstract

Though ursolic acid (UA) isolated from *Oldenlandia diffusa* was known to exhibit anti-cancer, anti-inflammatory, and anti-obesity effects, the underlying antitumor mechanism of ursolic acid was not fully understood to date. Thus, in the present study, the apoptotic mechanism of ursolic acid was elucidated in HCT116 and HT29 colorectal cancer cells in association with STAT3 and microRNA-4500 (miR-4500) by MTT assay, Terminal deoxynucleotidyl transferase-dT-mediated dUTP nick end labelling (TUNEL) assay, cell cycle analysis, immunofluorescence, and Western blotting. Ursolic acid significantly exerted cytotoxicity, increased TUNEL positive cells and sub-G1 apoptotic portion, induced cleavage of poly (adenosine diphosphate-ribose) polymerase (PARP) and caspase 3 in HCT116 and HT29 cells. Of note, ursolic acid attenuated the expression of anti-apoptotic proteins such as Janus kinase 2 (JAK2) and signal transducer and activator of transcription 3 (STAT3) and also blocked nuclear translocation of STAT3 in colorectal cancer cells. Notably, ursolic acid increased the expression level of miR-4500 in HCT116 cells by qRT-PCR analysis and conversely miR-4500 inhibitor reversed cytotoxic, anti-proliferative, and apoptotic effects by increasing TUNEL positive cells, PARP cleavage and inhibiting p-STAT3 in ursolic acid treated colorectal cancer cells. Overall, our findings provide evidence that usolic acid induces apoptosis in colorectal cancer cells partially via upregulation of miR-4500 and inhibition of STAT3 phosphorylation as a potent anti-cancer agent for colorectal cancer therapy.

## 1. Introduction

Colorectal cancer is one of the leading causes of cancer related deaths worldwide [1]. Several oncogenic molecules are involved in the progression of colorectal cancers including signal transducer and activator of transcription 3 (STAT3). STAT3, one of STAT family [2], is involved in cell growth and survival, inflammation, immune response, and cancer progression [3]. STAT3 dimerizes and enters into the nucleus by interacting with nuclear import proteins [4] through the activation of non-receptor protein tyrosine kinases such as Janus kinase1 (JAK1), JAK2, JAK3, epidermal growth factor receptor (EGFR) and SRC signaling [5,6]. MicroRNAs (miRNAs), small non-coding RNAs that reduce protein abundance and modulates several signaling pathways, are implicated in angiogenesis, proliferation, bone remodeling, and cancer progression [7,8]. Emerging evidence reveals that some miRNAs play important roles as oncogenes or tumor suppressors [9,10,11]. Hence, several natural compounds are attractive in the regulation of miRNAs in several cancers. Ursolic acid, a pentacyclic terpenoid that is derived from medicinal plants such as Oldenlandia diffusa, Mirabilis jalapa, and several fruits, is one of the antitumor compounds targeting oncogenic proteins and their related miRNAs. The miRNA 133a and miRNA21 are known as oncogenes in ursolic acid induced apoptosis in glioblastoma [12] and gastric cancer [13] cells, while miR-4500 acts as a tumor suppressor in cancer cells [14,15].

Additionally, though ursolic acid was known to induce apoptosis in several cancers such as lung cancer [16,17], breast cancer [18], prostate cancer [19], colon cancer [18], liver cancer [20], and melanoma [21] via phosphoinositide 3-kinase(PI3K)/AKT [22], EGFR/mitogen-activated protein kinase (MAPK) [23], p53 [24] and c-Jun N-terminal kinase (JNK) [25] pathways, the underlying mechanism of ursolic acid is not fully understood in colorectal cancers. Thus, in the current study, the pivotal roles of miR-4500 and STAT3 was investigated in ursolic acid treated colorectal cancers, since miR-4500 is partially associated with STAT3 sequence according to the miRWalk database (http://zmf.umm.uni-heidelberg.de/apps/zmf/mirwalk).

## 2. Results

### 2.1. Ursolic Acid Increased Cytotoxicity and the Number of TUNEL Positive Cells in HCT116 and HT29 Cells

**Figure 1:**
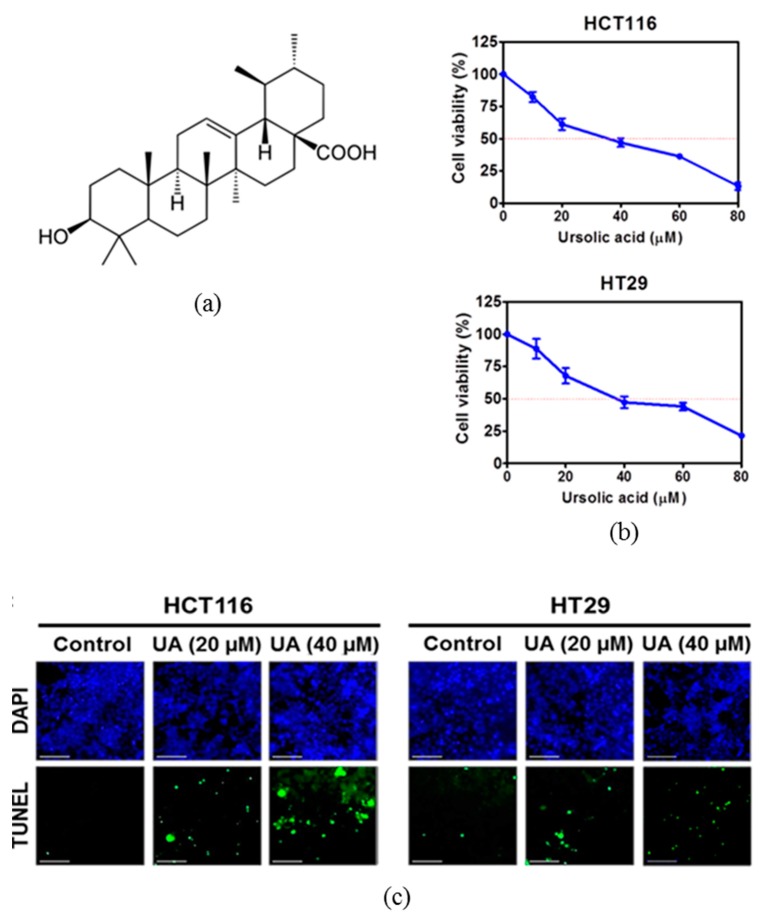
The effect of ursolic acid on cytotoxicity and terminal deoxynucleotidyl transferase-dT-mediated dUTP nick end labelling (TUNEL) positive cells in HCT116 and HT29 cells. (**a**) Chemical structure of ursolic acid. Molecular weight: 456.7 g/mol, molecular formula C30H48O3. (**b**) Cytotoxicity of ursolic acid was evaluated in HCT116 and HT29 cells. The cells were seeded into 96 well microplates and treated with various concentrations (0, 10, 20, 40, 60, or 80 μM) of ursolic acid for 24 h and then subjected to an MTT assay. (**c**) Effect of ursolic acid on the number of TUNEL positive cells in HCT116 and HT29 cells. Cells were treated with ursolic acid (0, 20, and 40 μM), and analyzed by TUNEL assay. The fluorescence microscopy was used to identify apoptotic TUNEL labeled cells (green), and DAPI stained cell nuclei (blue) by using a Delta Vision imaging system. Scale bar = 40 μm.

To evaluate the antitumor effect of ursolic acid (Figure 1a) in human colorectal cancer cells, MTT assay was performed. As shown in Figure 1b, ursolic acid showed significant cytotoxicity in a concentration-dependent manner in HCT116 and HT29 colorectal cancer cells. Visualization of DNA damage was observed by using terminal deoxynucleotidyl transferase-dT-mediated dUTP nick end labelling (TUNEL) staining, which is an assay for DNA break based on enzymatic labeling of free 3’DNA ends as one of the apoptosis features [26]. Treatment of ursolic acid significantly increased the number of TUNEL positive cells in HCT116 and HT29 cells as compared to the untreated control (Figure 1c).

### 2.2. Ursolic Acid Increased Sub-G1 Population and Regulated Apoptotic Proteins and Attenuated the Phosphorylation of JAK2/STAT3 in HCT116 and HT29 Cells

**Figure 2:**
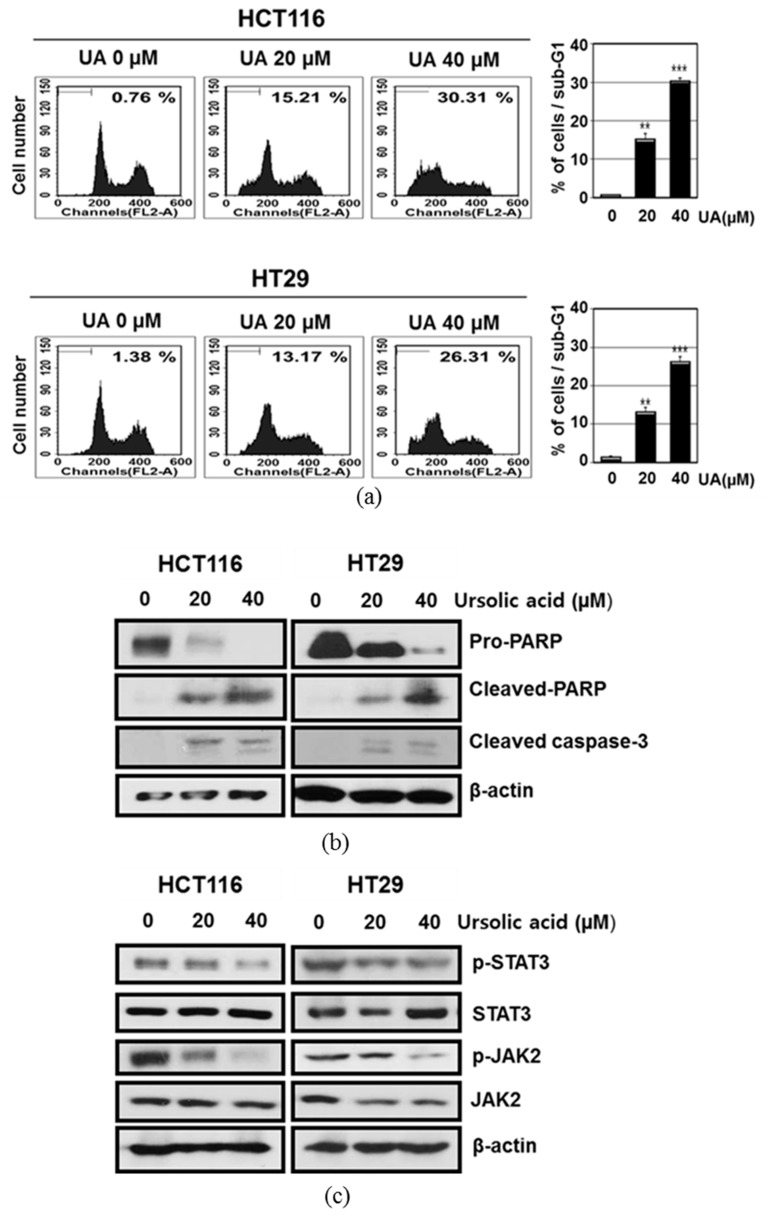
Effect of ursolic acid on the sub-G1 population and poly (adenosine diphosphate-ribose) polymerase (PARP), caspase 3, Janus kinase 2 (JAK2) and signal transducer and activator of transcription 3 (STAT3) in HCT116 and HT29 cells. (**a**) Effect of ursolic acid on sub-G1 population in HCT116 and HT29 cells. HCT116 and HT29 cells were treated with ursolic acid (20 and 40 μM) for 24 h and stained with propidium iodide (PI). Flow cytometric analysis was conducted for the sub-G1 apoptotic portion in HCT116 and HT29 cells. Bar graphs represent the percentage of sub-G1 DNA contents undergoing apoptosis. Data represent means ± S.D. ** *p* < 0.01, *** *p* < 0.001. (**b**) Effect of ursolic acid on the cleavages of PARP and caspase-3 in HCT116 and HT29 cells. HCT116 and HT29 cells were treated with ursolic acid (0, 20, and 40 μM) for 24 h. The cleavages of apoptosis-related proteins such as PARP and caspase-3 were measured by Western blot analysis. (**c**) Effect of ursolic acid on JAK2 and STAT3 signaling in HCT116 and HT29 cells. Western blotting was performed for p-STAT3, STAT3, p-JAK2, JAK2, and β-actin.

Apoptotic cells were measured as the percentage of the total cell population with sub-G1 DNA content after treatment with ursolic acid at doses of 0, 20, 40 μM for 24 h. The results showed that ursolic acid increased sub G1 population in a concentration-dependent manner in HCT116 and HT29 cells (Figure 2a). To investigate the apoptotic mechanism of ursolic acid, Western blotting was performed with apoptotic proteins in HCT 116 and HT29 cells. Western blotting revealed that ursolic acid cleaved poly (adenosine diphosphate-ribose) polymerase (PARP) and also significantly increased the expression of cleaved-caspase 3 in HCT116 and HT29 cells (Figure 2b). JAK-medicated tyrosine phosphorylation regulates the dimerization of STATs [27]. Herein, ursolic acid treatment dose-dependently attenuated the expression of p-JAK2 and p-STAT3 in HCT116 and HT29 cells (Figure 2c).

### 2.3. Ursolic Acid Blocked Nuclear Translocation of STAT3 in HCT116 Cells

**Figure 3:**
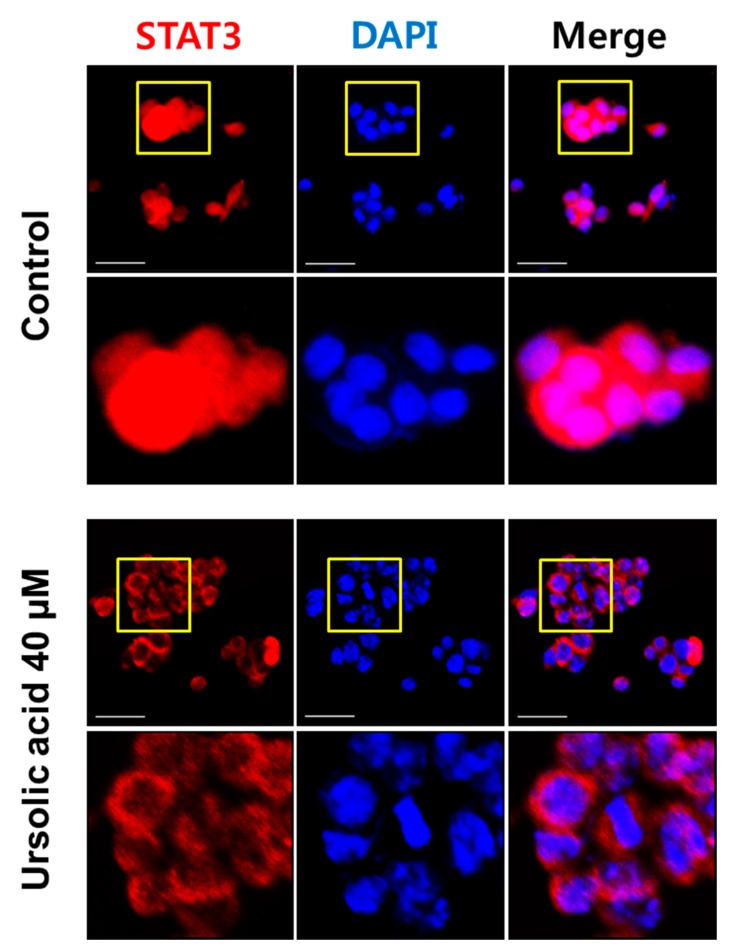
Nuclear translocation of STAT3 was suppressed by ursolic acid in HCT116 cells. The localization of STAT3 (red) and 4,6-diamidino-2-phenylindole (DAPI) (blue) in HCT116 cells. HCT116 cells were treated by ursolic acid for 24 h. STAT3 was probed with primary antibody and labelled using secondary antibody conjugated. Scale bar = 40 μm. Corresponding zoomed images of the STAT3, DAPI, and Merge (indicated by the yellow box).

STAT3 is activated by cytokines and growth factors via tyrosine phosphorylation (dimerization), and nuclear translocation [28]. Therefore, in order to investigate the nuclear trans-localization of STAT3, the immunofluorescence assay was used with STAT3 antibodies. As shown in Figure 3, the nuclear trans-localization of STAT3 was suppressed by ursolic acid in HCT116 cells.

### 2.4. Inhibition of miR-4500 Suppressed Cytotoxic and Anti-Proliferative Effects of Ursolic Acid in HCT116 and HT29 Cells

**Figure 4:**
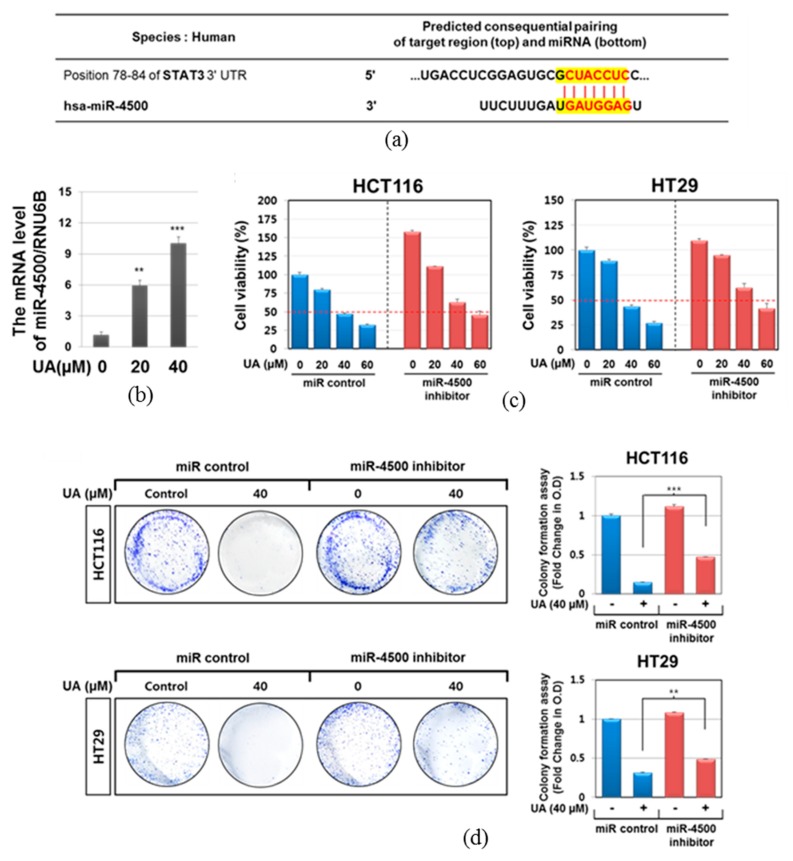
Down-regulation of miR-4500 attenuated cytotoxic and anti-proliferative effects of ursolic acid in HCT116 and HT29 cells. (**a**) Matched sequence (yellow box) of with mature miR-4500 and the STAT3. (**b**) Effect of ursolic acid on mRNA level of miR-4500 in HCT117 cells by qRT-PCR. (**c**) Effect of miR-4500 inhibitor on the cytotoxicity of ursolic acid in HCT116 and HT29 cells. The miR-4500 inhibitor and control plasmids were transfected into HCT116 and HT29 cells for 48 h and then exposed to ursolic acid for 24 h. Cell viability was determined by MTT assay. (**d**) Effect of miR-4500 on antiproliferative effect of ursolic acid by colony formation in HCT116 and HT29 cells for 2 weeks and colony formation assay was performed. ** *p* < 0.01, *** *p* < 0.001 vs. miRNA-4500 inhibitor negative control.

As shown in Figure 4a, miRWalk software (University of Heidelberg, Heidelberg, Germany) as a stringent bioinformatics approach predicts that sequence of miR-4500 partially matches to that of STAT3 (yellow highlighted sequence). Herein ursloic acid increased the level of miR-4500 in a dose dependent fashion in HCT116 cells (Figure 4b). To investigate the role of miR-4500 in cytotoxicity and apoptosis induced by ursolic acid in colorectal cancer cells. Inhibition of miR-4500 using miR-4500 inhibitor significantly reduced cytotoxicity by ursolic acid in HCT116 and HT29 cells compared to the untreated control (Figure 4c). Likewise, miR-4500 inhibitor reversed the reduced colonies by ursolic acid in HCT116 and HT29 cells two weeks after treatment (Figure 4d).

### 2.5. Critical Role of miR-4500 in Apoptotic Effect of Ursolic Acid in HCT116 Cells

**Figure 5:**
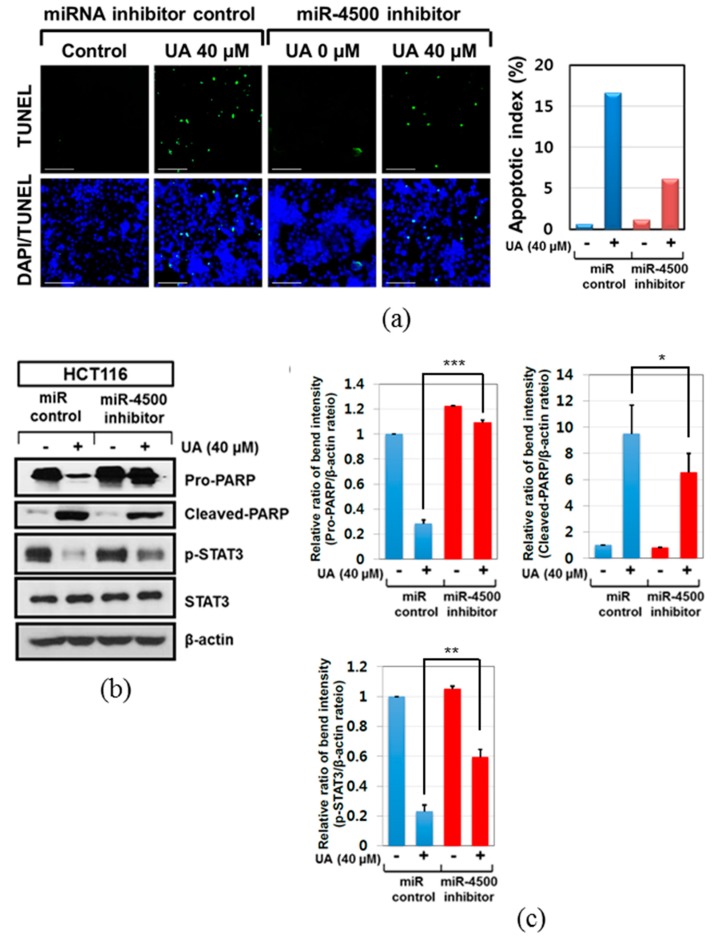
Critical role of miR-4500 in apoptotic effect of ursolic acid in HCT116 cells. (**a**) Effect of miR-4500 inhibitor on the number of TUNEL positive cells in ursolic acid treated HCT116 cells by TUNEL assay. Scale bar = 40 μm. Bar graphs showed quantification of TUNEL-positive cells (%). (**b**) Effect of miR-4500 inhibitor on PARP, p-STAT3, and STAT3 in ursolic acid treated HCT116 cells. (**c**) Bar graphs represent the relative expression of PARP or p-STAT3 to β-actin by using Image J software (https://imagej.nih.gov/ij/download.html). * *p* < 0.05, ** *p* < 0.01, *** *p* < 0.001 vs. miRNA-4500 inhibitor negative control.

To verify whether or not miR-4500 is critically involved in apoptosis and STAT3 inhibition by ursolic acid, miR-4500 inhibitor was transfected into HCT116 cells and treated with ursolic acid. As shown in Figure 5a, miR-4500 inhibitor was transfected into HCT116 cells and exposed to ursolic acid. TUNEL assay showed that the number of TUNEL positive cells by ursolic acid was significantly reduced HCT116 cells transfected by miR-4500 inhibitor. Consistently, Western blotting showed that miR-4500 inhibitor suppressed PARP cleavages and recovered the reduced phosphorylation of STAT3 by ursolic acid in HCT116 cells (Figure 5b). 

## 3. Discussion

Recently, natural compounds such as curcumin, decursin, ursolic acid, and others are attractive in several cancers as cancer chemo-preventive agents with fewer side effects [29]. In the current study, the underlying antitumor mechanism of ursolic acid was examined in association with miR-4500 and STAT3 signaling in HCT116 and HT29 colorectal cancer cells, since colorectal cancers comprise a group of molecularly heterogeneous diseases that undergo a variety of clinical courses and possess diverse therapeutic responses [30,31].

Herein, ursolic acid showed cytotoxic effects in a concentration dependent manner in HCT116 and HT29 colorectal cancer cells. To confirm whether its cytotoxicity is due to apoptosis in colorectal cancer cells, cell cycle analysis and TUNEL assay were performed. Ursolic acid increased the number of TUNEL positive cells and a sub-G1 apoptotic portion in colorectal cancer cells, implying the apoptotic potential of ursolic acid. However, there was not any significant difference by ursolic acid treatment in p53 null HCT116 cells and p53 mutant HT29 cells, possibly indicating a p53 independent pathway. Likewise, Pagliara et al. reported that 5-fluorouracil activated ribosomal protein L3 in HCT116 p53^−/−^ colon cancer cells as a proapoptotic factor [32], while mutant p53 confers chemoresistance in non-small lung cancer cells via upregulation of Nrf2 expression [33] or L3 downregulation [34].

Induction of apoptosis, so called programmed cell death [35], is generally accepted as an important strategy in cancer therapy. Western blotting showed that ursolic acid effectively induced cleavages of PARP and caspase-3 in HCT116 and HT29 cells, demonstrating the apoptotic effect of ursolic acid in colorectal cancer cells. Of interests, ursolic acid suppressed the phosphorylation of JAK2 and its downstream protein STAT3 in HCT116 and HT29 cells and consistently blocked nuclear translocation of STAT3.

There is accumulating evidence that some microRNAs are involved in carcinogenesis as oncogenes or tumor suppressors [7]. Though miR-4500 was known as a tumor suppressor in colorectal and non-small lung cancer cells [14,15], the underlying antitumor role of miR-4500 was never examined thus far in colorectal cancers by ursolic acid, though ursolic acid induces anti-cancer activity by targeting miR-21 [12], miR-133a [13], and miR-181a [36] in several cancer cells. In the current study, ursolic acid increased the expression level of miR-4500 in HCT116 cells by qRT-PCR. Of note, inhibition of miR-4500 using miR-4500 inhibitor reduced the apoptotic ability of ursolic acid to increase cytotoxicity and TUNEL positive cells and suppress the cleaved PARP and nuclear translocation of P-Jak2/p-STAT3 induced by ursolic acid in colorectal cancer cells, demonstrating the pivotal role of miR-4500 in apoptotic effect of ursolic acid via inhibition of Jak2/Stat3 signaling. However, it still needs further work, including animal study for future clinical trials, since these in vitro data are limited to completely validate the role of miR4500 and STAT3.

**Figure 6:**
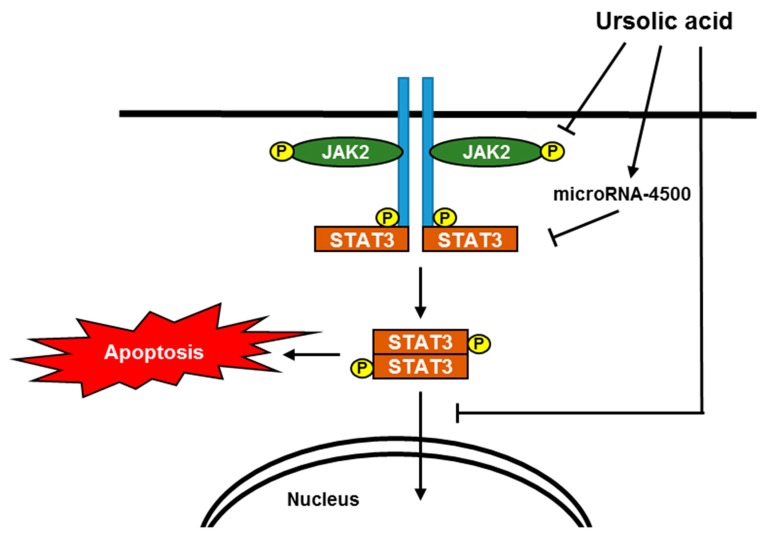
A schematic representation for the apoptotic mechanism of ursolic acid via upregulation of microRNA-4500 and inhibition of pJAK2/pSTAT3.

In summary, ursolic acid significantly increased cytotoxicity, sub-G1 population, TUNEL positive cells, and also cleaved PARP and caspase-3, and upregulated miR-4500 at mRNA level, and attenuated the phosphorylation of JAK2 and STAT3 in HCT116 and HT29 cells. However, miR-4500 inhibitor decreased cytotoxicity, colony formation, PARP cleavage, and p-STAT3 induced by ursolic acid in colorectal cancer cells. Collectively, these findings suggest that ursolic acid induces apoptotic effect via upregulation of miR-4500 and inhibition of STAT3 phosphorylation in colorectal cancer cells as a potent anti-cancer agent for colorectal cancer treatment (Figure 6).

## 4. Materials and Methods

### 4.1. Chemicals and Reagents

Ursolic acid (UA) (molecular weight: 456.7 g/mol, molecular formula C30H48O3) was purchased from Sigma (St. Louis, MO, USA). Also, antibodies specific for PARP, Cleaved PARP, Cleaved caspase-3, JAK2, p-JAK2, p-STAT3 and STAT3 (Cell Signaling, Beverly, MA, USA) and β-actin (Sigma Aldrich Co., St. Louis, MO, USA) were purchased for Western blot analysis.

### 4.2. Cell Culture

Colorectal cancer cells such as HCT116 (ATCC^®^ HTB-247™), HT29 (ATCC^®^ HTB-38™) were obtained from American Type Culture Collection (ATCC, Manassas, VA, USA). Cells were cultured in Roswell Park Memorial Institute medium (RPMI) supplemented with 10% fetal bovine serum (FBS) and 1% antibiotic (Welgene, Gyeongsan, Korea) at 37 °C in a humid condition of 5% CO_2_ for 24 h.

### 4.3. Cell Viability Assay

Cytotoxic effect of ursolic acid in HCT116 and HT29 cells was evaluated by using 3-(4,5-dimethylthiazol-2-yl)-2,5-diphenyltetrazolium bromide (MTT) assay (Sigma-Aldrich Corporation, St. Louis, MO, USA) according to the manufacturer’s instruction. In brief, the cells (1 × 10^4^ cells per well) were seeded onto a 96-well microplate and treated with various concentrations of ursolic acid (0, 10, 20, 40, 60, 80 μM) (Sigma, St. Louis, MO, USA) for 24 h. The cells were incubated with MTT (1 mg/mL) for 2 h at 37 °C in dark and MTT lysis solution (20% SDS and 50% dimethylformamide) was then added to each well. Then optical density (OD) was measured using a microplate reader (Molecular Devices Co., Silicon Valley, CA, USA) at 570 nm. Cell viability was calculated as a percentage of viable cells in the ursolic acid treated group versus the untreated control.

### 4.4. TUNEL Assay

To detect cell death, the DeadEnd™ Fluorometric TUNEL system kit (Promega, Madison, WI, USA) was used according to the manufacturer’s instructions (Roche Molecular Biochemicals, Mannheim, Germany). In brief, HCT116 or HT29 cells were treated with ursolic acid for 24 h and then washed with cold PBS. The cells were fixed with 4% paraformaldehyde for 30 min and washed twice with PBS for 2 min. Fixed cells in permeabilization solution (0.1% Triton X-100 and 0.1% Sodium citrate) were washed and incubated with TUNEL assay mixture for 60 min. The TUNEL-stained cells were visualized by a Delta Vision imaging system (Applied Precision, Issaquah, WA, USA).

### 4.5. Western Blotting

HCT116 or HT29 cells were exposed to ursolic acid for 24 h and were lysed in RIPA buffer (50 mM Tris-HCl, 150 mM NaCl, 2 mM EDTA and 1% Triton X-100) containing phosphatase inhibitors (Sigma, Saint Louis, MO, USA) and protease inhibitors (Roche, Mannheim, Germany). The protein samples were separated on 8% to 15% sodium dodecyl sulfate-polyacrylamide gels (SDS-PAGE) and were transferred to nitrocellulose membranes. Membranes were incubated with primary antibodies of PARP, cleaved PARP, cleaved caspase-3, JAK2, p-JAK2 (#3771), p-STAT3 (#9131) and STAT3 (Cell Signaling, Beverly, MA, USA) and β-actin (Sigma Aldrich Co., St. Louis, MO, USA). These were diluted in 3% bovine serum albumin (BSA) and in PBS-Tween 20 (1:500–1:2000) at 4 °C overnight, washed three times with PBS-Tween20 and finally incubated with HRP-conjugated secondary antibody (1:2000). The expression was visualized by using ECL Western blotting detection reagent (GE Healthcare, Amersham, UK).

### 4.6. Sub-G1 Accumulation by Cell Cycle Analysis

Cell cycle analysis was performed by propidium iodide (PI) staining. HCT116 or HT29 cells were treated with Ursolic acid for 24 h and collected and fixed in 70% ethanol. The cells were then incubated at 37 °C with 0.1% ribonuclease A in PBS for 30 min and suspended in PBS containing 30 μg/mL PI for 30 min at room temperature. Sub-G1 accumulation was evaluated from the stained cells by FACS Calibur (Becton Dickinson, Franklin Lakes, NJ, USA) using the Cell Quest program (Becton Dickinson, Franklin Lakes, NJ, USA).

### 4.7. Immunofluorscence

Colorectal cancer cells treated with ursolic acid were fixed with 4% formaldehyde and then permeabilized in 0.1% Triton X-100. The fixed colorectal cancer cells were then washed with 1× PBS and blocked with 2% bovine serum albumin (BSA) in 1× PBS for 30 min at room temperature. Fixed cells were incubated with the specific primary antibody of STAT3 antibody (Cell signaling, Boston, MA, USA) overnight at 4 °C. After washing, the cells were incubated with Alexa Fluor 546 goat rabbit-IgG antibody (Life technologies, Waltham, MA, USA) (1:1000) for 1 h at room temperature. After washing twice, the nuclei of the cells were stained with 4,6-diamidino-2-phenylindole (DAPI; Sigma, Saint Louis, MO, USA). Images of STAT3 and DAPI stained cells were taken by a Delta Vision imaging system (Applied Precision, Issaquah, WA, USA).

### 4.8. RNA Isolation and Quantitative Real Time Polymerase Chain Reaction (qRT-PCR)

Total RNA was extracted from HCT116 cells that were exposed to ursolic acid (0, 20, and 40 μM) for 24 h by using the ReliaPrep RNA Cell Miniprep System (Promega Corp., Z6010, Madison, WI, United States) and one microgram of total RNAs was used to make cDNA by superscript reverse transcriptase and amplified by Platinum Taq polymerase with Superscript One Step RT-PCR kit (Invitrogen, Carsbad, CA, USA). Primer sequences used synthesized by HB Nucleic mix (Heimbiotek, Gyeonggi-do, Korea). For PCR amplification, following steps were undertaken; an initial step at 50 °C for 30 min, 94 °C for 2 min, followed by 30 cycles at 94 °C for 15 s, 55 °C for 30 s and 72 °C for 1 min, and a final step at 72 °C for 10 min. Then RT-qPCR was performed with HB Real-Time PCR master mix kit (Heimbiotek, Gyeonggi-do, Korea).

### 4.9. RNA Isolation and Quantitative Real Time Polymerase Chain Reaction (qRT-PCR)

Total the miR-4500 inhibitor and miRNA control from Bioneer (Daejeon, Korea) were transfected into colorectal cancer cells using X-treme transfection reagent (Roche Applied Biosystem, Basel, Switzerland) according to the manufacture’s protocol. Two days after transfection, colorectal cancer cells were treated by ursolic acid for 24 h and then were harvested for cell viability assay, colony formation assay, TUNEL assay, and Western blotting.

### 4.10. Statistical Analysis

Data were presented as means ± standard deviation (SD). The statistically significant differences between control and ursolic acid treated groups were calculated by Student’s *t*-test. *p* value < 0.05 was considered statistically significant. All experiments were carried out at least three times.

## Abbreviations

PARP	Poly (ADP-ribose) polymerase
Caspase	Cysteine aspartyl-specific protease
STAT3	Signal transducer and activator of transcription 3
JAK2	Janus kinase 2
mRNA	Messenger RNA
